# Abusive Practices, Self-regulation Strategies, and the Language of Addiction: Narratives Surrounding Problematic Smartphone Use in Southeastern Spanish Youth

**DOI:** 10.1007/s11013-025-09964-x

**Published:** 2026-02-09

**Authors:** José Palacios Ramírez, Joaquín Rodes García, Antonio M. Nogués Pedregal

**Affiliations:** 1 Miguel Hernandez University, Elche, Spain; 2 Universidad Católica San Antonio de Murcia, Murcia, Spain

**Keywords:** Youth, Smartphone, Abuse practices, Self-regulation strategies, Culture of addiction

## Abstract

The smartphone has become fundamental in areas, such as leisure, sociability, and intimacy, especially for young people. Its impact has led to growing concerns about its effects, in terms of a potential addictive nature. This article approaches the articulation of abuse and self-regulation practices regarding the smartphone, and the presence of addictive explanations as a framework that links such practices to a culture of addiction. For this purpose, 24 young people (between 18 and 29 years) with different socio-demographic profiles (age, gender, occupation) and levels of problematic use of digital technologies were interviewed. The results show how respondents regulate the constant influx of smartphones into their daily lives with individual strategies that attempt to balance the perceived effects of their digital practices. Their effectiveness is modulated by aspects, such as maturity, personal context, or commitment to other activities. In many cases, their self-perceptions are permeated by popularized discourses of addiction, which coexist with ironic positions regarding their agency facing the technological designs and are aligned with logics of individual responsibility. They also highlight emergent aspects, such as the link between weekend binge or nightly practices of smartphone abuse, with their own personal sphere and self-gratification.

## Introduction

The smartphone has become the core device of digital technologies, due to its capacity to interconnect the digital transformation (Fussey & Roth, 2020, p. 660; Trittin-Ulbrich et al., 2021, p. 10) and the updating of individualization processes, a key axis in the development of modern societies (Martin, 2007; Elias, 2015; Jiang et al., 2018). The potential to be integrated at a bodily level (Sadin, 2017) has placed it at the center of everyday rituals that intertwine contemporary trends, such as personalized consumption, new forms of sociability, and the redefinition of intimacy (Ling, 2013; Koren et al., 2015).

In the process of increasing its uses, the smartphone has undergone a rapid recreational evolution based on its capacity to re-mediatise multimedia content and formats (Cunningham, 2015; Fullwood et al., 2017; Krotz, 2022). This evolution has paralleled positioning it as an integrator of different forms of presence (Knorr Cetina, 2009; Jameel et al., 2019), straining contemporary sociability with complex implications on the shaping of subjectivity (Kirmayer et al., 2013; Childs & Holland, 2022). These range from the updating of youth peer groups (Goggin, 2013; Lim, 2015), to the redefinition of romantic relationships (Garris, 2020).

Therefore, it is understandable why, behind apparently banal arguments about the intensive use of the smartphone, such as *escaping boredom* or *keeping in touch* (Amiri & Dowran, 2020; Alotaibi et al., 2022), meanings of identity and emotions can be perceived that are denser than presumed (Irimiás et al., 2021). At the same time, there has been a growing social concern about what is perceived as widespread abuse, expressing concern about the possible negative effects of rapidly expanding digitalisation processes. This negative social perception primarily covers matters such as time management, attention span, and the effects on social interactions. But in a deeper sense, it involves the future projection of concerns and hopes in the face of a horizon of still uncertain structural changes (Escobar, 1995; Rabinow et al., 2008).

### Smartphone abuse: between problematic use and addiction

There is a striking contrast between the scientific and social interests sparked by the different aspects of the smartphone. While there is little question about issues such as the ecological impact or labor exploitation (Miller et al., 2021, p. 22), the interest in excessive use, especially among young people, has increased exponentially. This true example of interactive dynamics (Hacking, 2007) between scientific research and social concerns, has crystallized in the contraposition of problematic use and addiction. Understanding addiction as a dysfunctional craving with associated psychosocial and functional impairments (Kuss & Griffiths, 2017) and problematic use as a pattern of excessive use with negative effects (Pearson & Hussain, 2015; Li & Lin, 2019; Yang et al., 2021; Arrivillaga et al., 2022; Kim, 2022; Rodríguez-Torrico et al., 2023). This opposition includes abuse and patterns of addictive behaviour as intermediate stages (Lapointe et al., 2013; Domoff et al., 2019; Dai et al., 2021).

While the concept of problematic use has gained greater clinical acceptance, as there are hardly any cases of addiction described in Western contexts, it remains unclear whether these are distinct conditions or levels of severity on the same continuum. In any case, regardless of the similarities with conventional addictions (Billieux et al., 2015, pp. 157–158), from a social point of view, the most relevant aspects would be the psychosocial profiles noted by some studies (Billieux, 2012), and the problems of emotional management, stress, or personal dissatisfaction that can apparently be channelled through smartphone abuse (Freitas et al., 2022, p. 5; Marino et al., 2022; Shi et al., 2023).

This lack of consensus has led to the notion of smartphone addiction being used loosely to describe problems associated with digital technologies (Kuss & Griffiths, 2012). Aside from epistemological issues, such as the explanatory hierarchy of the biopsychosocial components of behavior (Meloni, 2014) or limitations in scientific evidence regarding community or individual approaches (Kristjansson, 2019; Olson et al., 2022), the effects of the addiction framework have quickly shifted from ambivalence to potentially negative connotations. The medicalization of youth digital practices (Van Rooij & Prause, 2014; Kardefelt-Winther et al., 2017; Panova & Carbonell, 2018) promoted by the educational and health sectors as well as corporate strategies of the digital industry itself, could be placing the focus on rare severe cases mediated by psychosocial issues. This denies a debate on widespread abusive practices, which extend beyond young people. Furthermore, this minimizes the structural significance of persuasive design and attention-grabbing strategies embedded in digital technologies (Fogg, 2003; Williams, 2018).

It may be true that young people are as addicted to smartphones as alcoholics are to their bottles (Kuss & Griffiths, 2017). However, the consumption format should not be disregarded (Becker, 1971; Antze, 1987) as part of the symbolic framework, through which we learn to perceive and assess the effects of alcohol or the smartphone. In this sense, it is interesting to consider the specific role of the smartphone in the practice of ‘digital commensality.’ This is suggested by studies on patterns of use among parents (Bae & Nam, 2023), family regulation systems (Teichert, 2020), correlation with reading habits (Levratto et al., 2021), or age of access to the first smartphone (Gerosa & Gui, 2023; Perowne & Gutman, 2023).

### The question of smartphone abuse and the onset of a culture of addiction

The social status of the concept of addiction (Raikhel et al., 2013, p. 1) is due to its ability to assemble networks of biomedical knowledge and technologies, cultural environments, and forms of subjectivity. These assemblages shape realities that are problematized (Rabinow, 2003, p. 20) in alignment with the economic-political and cultural conditions of a context, as is the case with the current problematization of smartphone misuse.

From this perspective, the difficulties of expert approaches establishing objective empirical indicators of the problem are striking. This has led to an “instrumental” interest in qualitative research that exclusively seeks to contrast quantitative and psychological models (Clayton et al., 2015; Tossell et al., 2015). Thus, the explanatory deficit of many qualitative analyses may be related to their unquestioning of the sociocultural background of the perceptions on which they are empirically based. The social influence of the concept of addiction should be considered in this area.

The popularization of the concept of addiction as an explanatory framework is embedded in the extension of the behavioral model of addiction (Leshner, 1997) and the “cultural turn” it implies (Shelby, 2016, pp. 79 ff.; Maarefvand & Ghiabi, 2024; White, 1996). Previously, the concept of addiction delimited entities that condensed labeling processes of problematic subjects together with languages of social suffering (Kushner, 2010, pp. 20 ff.; Sedgwick, 1992; Courtwright, 2001). But with the behavioral turn and in line with a liberal governmentality of addiction (O’Malley & Valverde, 2004), this framework is decentered. Labeling mechanisms reach socially integrated sectors and behaviors (Vrecko, 2010, p. 40), and as an expressive language, addiction is extended to subjective discomforts linked to a struggle for self-control and well-being (Le Breton, 2016; Martínez Hernáez, 2020).

This emerging addiction regime maintains some previous aspects, such as individual responsibility (Rose, 1999). However, it also introduces new logics, such as the epidemiological redefinition of addictive behavior (Espósito, 2005) or the interplay between the democratization of risk practices and the liberalization of the therapeutic assistance network (Schüll, 2013). The result is a kind of “addiction culture” that not only normalizes intensive practices that can become problematic but also challenges individuals to constantly problematize the potential addictiveness of their daily habits.

These issues are often viewed with suspicion by digital anthropology, which prefers to focus on the ambivalence and potential of digital technologies. From this perspective, concerns about the effects of the abuse of these technologies evoke a romanticization of the pre-digital world and Protestant notions of authenticity (Horst & Miller, 2012). However, recent ethnographic research on digital detoxification communities or therapeutic groups (Sutton, 2020; Tulasiewicz, 2024) is starting to focus on how, beyond clinical considerations, certain sectors are questioning the potential social harms associated with the misuse of these technologies.

With regard to the use of smartphones and other digital technologies, the effects of this “addiction culture” are manifested in the deployment of a therapeutic and detoxification offer parallel to standardized diagnoses. Or in the expansion of the rhetoric of addiction in the media, exemplified by the personal stories of celebrities (Travis, 2010; Tiger, 2013). But more visibly in the efforts that individuals make to self-regulate their digital practices, and in the presence in their narratives of what some authors call the cultural language of addiction (Raikhel, 2015, p. 340; Vrecko, 2010).

This concern about the effects of smartphone misuse present a global dimension (Collier & Ong, 2004). In some cases (Adorjan & Ricciardelli, 2021), studies show that the label of addiction is adopted by young people alongside what they perceive as low agency in the face of technological designs. In others (Freitas et al., 2022; Shi et al., 2023), the notion of addiction is linked to perceptions of smartphone use aimed at achieving emotional regulation. In addition, some studies, although not explicitly using the term addiction, report that participants continue to abuse their smartphone use despite perceived negative effects (Amiri & Dowran, 2020) or have to make significant efforts to maintain self-regulatory practices (Dai et al., 2021).

Something similar happens in the case of the digital practices of Spanish youth. Although many studies try to avoid contributing to the labeling of young people as a problematic group, the data show concern about digital misuse, although not always in terms of addiction. In some studies, participants admit moments of lack of control (Orgaz Alonso et al., 2024) or express an excessive use of the smartphone (Megías, 2024). Others point to the possibility of compulsive and risky use among the most socioeconomically vulnerable populations (Gómez-Miguel & Calderón-Gómez, 2022). And, of course, there are studies in which participants express the perceived risk of addiction (Castañeda et al., 2019; Torrijos-Fincias et al., 2021) or define themselves as addicts (Hoz, 2018).

This paper addresses the articulation of abusive and self-regulatory practices surrounding the smartphone, and their narrative presentation within the framework of what we call a culture of addiction. Based on semi-structured interviews with young people from the southeast of Spain, with different socio-demographic profiles and levels of digital technology abuse, we analyze their perceptions of their abuse and self-regulation practices, as well as their use of explanatory metaphors related to the idea of addiction. The main aim is to analyze transversal narrative patterns related to the pursuit of balance in digital use, in a context where the presence of digital technologies is intense and there is growing social concern about their possible effects.

Typically, the implications of abuse of digital technologies are analyzed from a psychological perspective, while sociocultural approaches tend to focus on the role of the smartphone in young people’s digital practices. However, it is interesting to consider the practical ways in which young people manage the complex trade-offs between abuse and regulation in digital practices focused around their smartphones (Adorjan & Ricciardelli, 2021), as well as the role that addictive explanations plays in the construction of these practices (Raikhel et al., 2013).

## Methodology

### Design and procedure

This study is part of a wider research into the problematic use of digital technologies among young people in the city of (Spain). The methodological design of the study included two distinct phases. The first involved a quantitative exploration, surveying a sample of 431 young people aged between 15 and 29 whose representativeness was adjusted to initial quotas of sex, age, and neighborhood according to the Registro Municipal de Habitantes (Municipal Population Register). The questionnaire included three modules: a socio-demographic profile, a section on the use of digital technologies and the Compulsive Internet Use Scale (CIUS). The Spanish Ministry of Health and the main national stakeholders in research on youth issues use this age group in many studies, and the Observatorio Nacional sobre Drogas (National Drug Observatory) also uses the CIUS scale to estimate the abuse of digital technologies (Observatorio Español de las Drogas y las Adicciones, 2023). This scale has been validated and used in different contexts to analyze aspects of digital abuse practices linked to Internet use (López-Fernández et al., 2019).

The second phase focused on conducting semi-structured interviews. To this end, we recruited respondents from the survey who agreed to be interviewed. This process resulted in 15 of the 24 interviews that make up the study, and the rest were obtained through a snowballing procedure with the interviewees. The participants recruited through this procedure had previously completed the same survey. Most of the interviews were conducted face to face, using places arranged with the interviewees or a space provided by the university. In some specific cases, the interview was conducted by videoconference at their request. In all cases, the interviewees were briefed on the research orientation and their right to leave at any time, and they also signed an informed consent form.

The interview script was organized into five blocks: a section on socio-demographic characteristics (age, gender, employment, and housing situation); a section focusing on the uses of digital technologies with questions about their daily life (devices, applications, and functions); a section on digital abuse practices; a section focusing on the sensations experienced during these practices; and a final section that gathered opinions on the possibility that the abuse of digital technologies could constitute an addiction.

### Sample

The sample of young people interviewed (see Table 1) reflects a range of socio-demographic characteristics (gender, age, employment, and housing situation), and their level of risk of problematic use of digital technologies (score on the CIUS scale). The sample of 24 interviewees consisted of 13 boys and 11 girls. Of these, 11 (5 boys and 6 girls) were in what was established as the upper age group (25–29 years), 10 (6 boys and 4 girls) in the middle age group (20–24 years), and 3 (2 boys and 1 girl) in the lower age group (15–19 years). In terms of employment, 16 of the interviewees were studying at university (2 were combining study with work), 7 were working, and 1 was preparing for civil service exams. A total of 11 did not live with their parents, and of these, 2 shared housing (as they were studying in another city). In terms of the risk of problematic use, three levels were established that grouped the scores obtained on the CIUS scale into low risk (up to 18 points), medium risk (between 19 and 34 points), and high risk (between 35 and 56 points). Of those interviewed, 8 (5 boys and 3 girls) obtained high-risk scores, 10 (5 boys and 5 girls) medium-risk scores, and 6 (3 boys and 3 girls) low-risk scores. The criterion for this grouping was to stress the differences between the risk profiles at the extremes (high and low).

**Table 1:** Participants in the research interview

Interview code	Gender	Age group	Risk level	Housing	Occupation
Julia	Female	25 High	High	Shared	Studying
Verónica	Female	25 High	Low	On her own	Studying and working
Rafael	Male	28 High	Low	On his own	Working
Jordi	Male	23 Medium	Medium	With his parents	Studying
Sabrina	Female	21 Medium	Medium	With her parents	Studying
Raúl	Male	25 High	Medium	On his own	Working
María S.	Female	25 High	Medium	With her parents	Studying
María B.	Female	28 High	Low	With her parents	Civil servant exam
Jose David	Male	25 High	Low	On his own	Studying
Jorge	Male	25 High	Medium	On his own	Working
Joaquín	Male	23 Medium	High	On his own	Studying and working
Iván	Male	23 Medium	Medium	Shared	Studying
Illen	Female	29 High	Medium	On her own	Working
Diana	Female	25 High	Medium	On her own	Working
Daniela	Female	22 Medium	High	With her parents	Studying
Carla	Female	21 Medium	High	With her parents	Studying
Basilio	Male	21 Medium	High	With his parents	Working
Antonio	Male	19 Low	High	With his parents	Studying
Javier	Male	22 Medium	Medium	With her parents	Studying
Alejandro	Male	22 Medium	Low	On his own	Studying
Marcelo	Male	18 Low	High	With his parents	Studying
Mariana	Female	23 Medium	Medium	With her father	Studying
Estefanía	Female	19 Low	Low	With her parents	Studying
Ignacio	Male	25 High	High	With his parents	Working

Authors’ elaboration

### Data analysis

A thematic analysis was performed using the MaxQDA software (version 4.01.2022). This analysis aimed to identify recurring patterns of meaning across the data (Braun & Clarke, 2006), employing a step-by-step process from transcription to coding and conceptualisation (Naeem et al., 2023). The interviews were transcribed and prepared (punctuation, significant pauses) in order to take advantage of the analytical tools of this software. The analysis was structured following phases of exploration, coding, and relativisation (Strauss, 1987; Taylor & Bogdan, 1998). The coding system was developed collaboratively among the authors (Kuckartz, 2014), starting from the initial coding of a small sample of interviews according to a theme-based strategy. The analysis was mainly inductive, based on codes and categories that allowed for the comparison of cases, framing them within the study variables. Nevertheless, some in-vivo codes emerged during the analysis process as a result of the work done at word level using the Maxdictio tool. These emerging codes allowed us to reorientate the relationship between the categories and the refinement of the conceptual framework.

From the initial outline of the study, centered on the abuse of digital technologies in general, the codes ‘abuse practices’ and ‘self-regulation strategies’ were generated. At first, the code abuse practices included several sub-codes, such as ‘sensations during intensive use,’ ‘digital immersion,’ or ‘presence of conflicts.’ The code control strategies was not structured into sub-codes. However, the emergence of the smartphone code led to a substantial reordering of this scheme.

Despite including some specific questions on preferences and the most frequent uses of devices, the interviews were not initially intended to specifically explore smartphones. After noting the abundance of references to the smartphone, the most used term with 606 references, ahead of social networks (216) and Instagram (184), we proceeded to create a dictionary of associated terms (mobile phone, mobile phones, telephone...) and labeled it with the category ‘smartphone.’ This code turned out to be by far the one with the most coded fragments (311), including fragments from the 24 interviews (100% coding frequency). There was also another relevant indication, such as a significant concurrence (50%) of fragments coded with the codes ‘abuse practices’ and ‘self-regulation strategies.’ To which we should add the observation that, even when interviewees are talking about social networks or streaming, they are actually still talking about smartphone use. The emergence of the ‘smartphone’ code, in turn, allowed for a new sub-coding of ‘abuse practices,’ which came to be called ‘time as a perceived marker of abuse’; ‘the smartphone as a distraction’; and ‘smartphone use before going to sleep.’

Something similar happened with the code ‘language of addiction.’ The interviews collected the perceptions of the participants about the possible existence of an addiction to digital technologies, and the distribution of responsibilities between design and individual usage patterns. But the analysis revealed a large number of fragments referring to the relationship of the interviewees with the smartphone from 70% of the interviews, and with a significant concurrence of coding with respect to the code ‘abuse practices.’ Therefore, the procedure described was followed with Maxdictio (hooked; addicted; addiction; cravings; withdrawal; addictive). The addiction language code also includes several sub-codes: ‘addiction to digital technologies and its causalities’; ‘the language of addiction applied to others’; and ‘the language of addiction applied to oneself’ (Table 2).

**Table 2:**
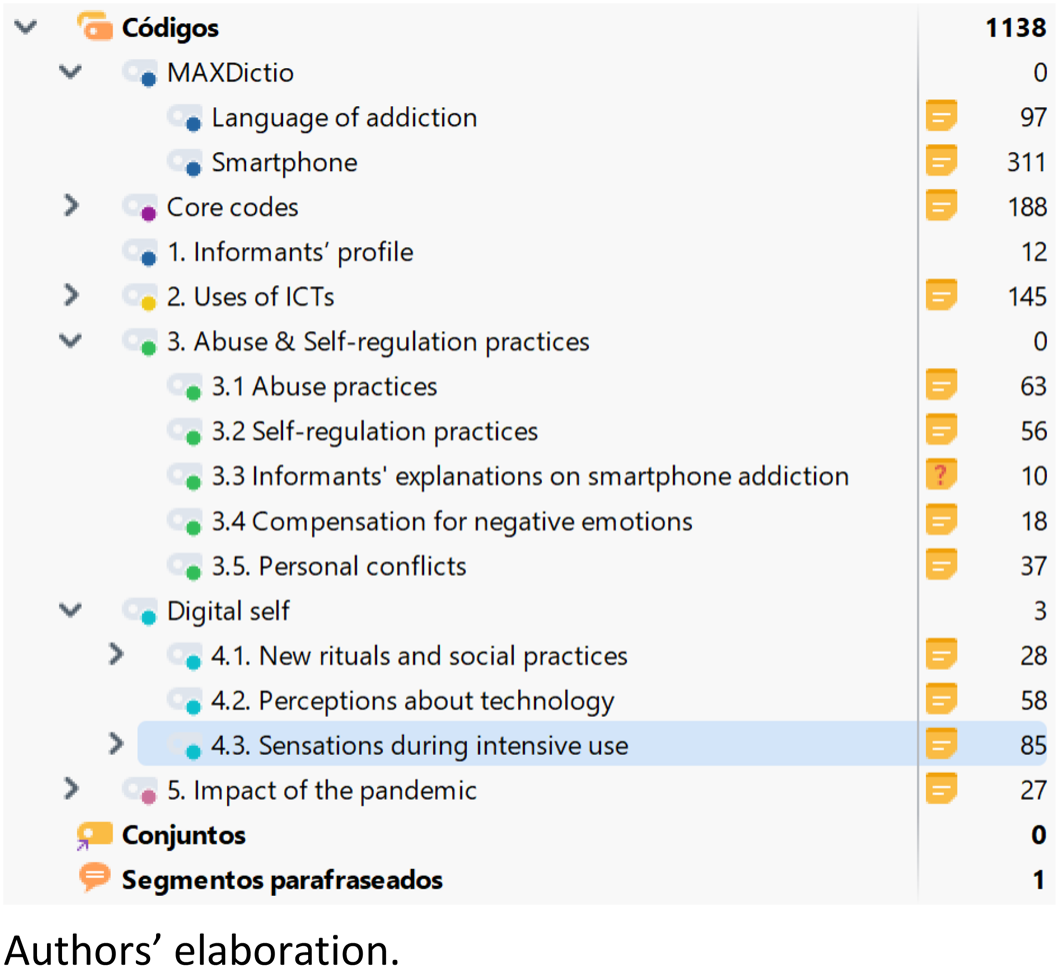
Research codes count

***Ethical criteria***

This study was approved by the research ethics committee of the Universidad. The committee, having evaluated the characteristics and ethical documentation related to the project, issued a favorable report.

## Results

Despite the socio-demographic differences and variations in the CIUS scale scores between the participants, the digital practices described in the narratives can be understood as a relatively coherent set. Only the level of risk seems to be a relevant differentiating factor, especially when comparing the high- and medium-risk groups with the low-risk group. Meanwhile, age seems to have an influence in some specific aspects. In this sense, we must underline the need for a dynamic understanding of both variables. Age can probably be better understood as a rough estimate of the degree of maturity and the level of obligations or time available for leisure activities. Whereas the level of risk of problematic use ultimately corresponds to the perceptions expressed by the interviewees at a specific moment in time, which possibly leads one to imagine the same interviewee in other risk groups depending on their personal context.

As previously mentioned, the interviewees share some fairly common usage patterns. The smartphone acts as a hub for digital activity, becoming deeply embedded in daily routines, which produces an influence that most people try to control with varying degrees of success. Communication applications (WhatsApp), social networks (Instagram, TikTok, and YouTube) and scrolling are the practices that feature most prominently and capture the attention of a large proportion of the interviewees. Which in turn places them at the center of self-regulation practices and the problematization of potential addictiveness. Nevertheless, it is true that this pattern fades somewhat as the level of risk decreases, giving way to the use of other devices, such as computers and other applications related to streaming (Spotify), where use interferes less with daily activities or is limited to a more conventional definition of leisure time.

### Abuse practices

The ‘abuse practices’ code covers everyday smartphone use related to the impact of excessive use. As mentioned above, the code was organized as follows: ‘time as a perceived marker of abuse’; ‘the smartphone as a distraction’; and ‘smartphone use before going to sleep.’ The intensity of these practices is generally distributed based on the risk of problematic use, although age can have a moderating effect.

## Time as a perceived marker of abuse

The use of screen time as an indicator of abuse is relatively common, especially among members of high-risk groups (Joaquín, Julia, Daniela) and medium-risk groups (Diana, Illen, Mariana). The references to the subject relate a futile struggle to self-impose a time of use which, despite often being exceeded, provides a sense of control.

*(Interviewer): And how did you attempt to do it? Did you succeed? Did you fail?*

*(Mariana): Well, sometimes I succeed and sometimes I don’t. (Mariana, medium-risk).*

Unlike the rest, most of the high-risk group (6 out of 8) offered definite timings (Antonio 9 h; Basilio 6 h; Carla 5 h; or Daniela 10 h). In the low-risk group, only 2 of the 6 interviewees (Estefanía and José David) understood that exceeding a certain amount of time was a marker of abuse. For example, María B. mentioned that she usually checks the weekly usage time and that it bothers her if it is too much.

*(María): [Hum], It’s something that-that bothers me, and it’s like a thing that I don’t really want to spend a lot of time on. I mean, I don’t like wasting, not wasting, I mean, I don’t like spending my time, on-on being in front of the screen. (María B., low risk).*

## The smartphone as a distraction

The depiction of the smartphone as an element that interferes with everyday activities is widely reflected in the narratives of the high- and medium-risk groups (only 2 references in the low-risk group), with the distinction that among the high-risk group, it is perceived as a distraction, and among the medium-risk group, it is perceived as a temptation.

In the high-risk group, Basilio (also Antonio) refers to the power of the smartphone in this sense: ‘without an external factor that makes you escape, it is difficult to resist.’ While others, such as Marcelo (also Ignacio, Carla), describe the distorting effect of the smartphone on activities such as studying.

*(Marcelo): No, what happens is that if I’m in one of those moments of being absent-minded with my phone in my room and I’m supposed to be studying, if I hear footsteps in the corridor, I get nervous and I panic, thinking they’re going to open the door and find out that I’m slacking off and stuff like that. (Marcelo, high risk).*

This distraction appears to be affected by age in the medium-risk group*.* For those in the older age group (Diana, Raúl, and Jorge), the smartphone acts as a distraction in their free time, when doing things like watching television. Whereas for others in the middle age group (Mariana, Illen, and Jordi), smartphone temptation appears in ‘useful’ time, and as something they take preventative measures against. When referring to study time, Mariana switches it off and leaves it outside the room, switching it on ‘only during breaks’; Illen finds it difficult to study with the smartphone ‘nearby’; and for Jordi ‘the temptation is greater’ if he is bored. Meanwhile, Verónica, from the low-risk group, expressed an interesting differentiation: the smartphone can act as a distraction in class but not at work, as she is not allowed to use it there.

## The use of the smartphone before going to sleep

A striking element in the abuse practices is the nocturnal use of the smartphone. Once again, these practices predominate in the high- and medium-risk groups and are practically non-existent in the low-risk group (Verónica). Contrary to expectations, only 3 members of the high-risk group reported feeling extra tired as a result of this nocturnal use. Iván and Illen (both medium risk) or Verónica (low risk) report seeing content in bed before sleeping without this causing them any problems. Unlike Antonio (high-risk), who recognized the cost in tiredness: ‘you feel good but then it takes its toll.’

As for the possible impact on sleep, cases such as Carla (high risk): ‘Without TikTok videos at night I can’t sleep,’ contrast with the statements of Diana and Raúl (both medium risk), who note that the feeling of immersion produced by night-time smartphone use makes it difficult for them to fall asleep.

*(Raúl): Yes, that’s happened to me. Look, actually last night, the same thing happened to me that—well, you sit down to watch a series and—and before you know it, you get hooked, and before you know it, it’s two in the morning. You end up going to sleep late, you end up—you end up getting up late and...’ (Raúl, medium risk).*

On the other hand, some interviewees such as Marcelo (high risk) and Mariana (medium risk) engage in this practice at other resting times, such as after lunch, during the ‘siesta’/nap time.

### Self-regulation strategies

Self-regulation strategies are opposed to smartphone abuse practices. These are strategies developed by the interviewees to modulate their abuse practices and create a more or less lasting sense of control. Similarly to abuse practices, transversal elements such as the individual nature of the strategies take precedence over the differences. Once again, the most operative variable is the level of risk, albeit in a different sense to what might be presumed, since it is in the high-risk groups (with 6 out of 8 members) and medium-risk groups (with 6 out of 10 members) where these strategies are most present (only 2 out of 6 low-risk members).

Beyond their distribution by risk group, two main types of strategy can be identified. The first includes more conventional strategies, based on specific and discontinuous interventions. The second involves more creative strategies, which often rely on digital technologies themselves. In the first group, Antonio (high risk) and Javier (medium risk) uninstall the app they spend the most time on during exam periods. Meanwhile, Joaquín (high risk), Diana, Jorge and Jordi (all medium risk), set limits on usage time using alarms on their smartphones, and although they recognize that they regularly overrun these limits, they find them useful in ‘keeping a degree of control.’ In the second group, the predominant approach is to make use of digital technologies. Julia (high risk) tries to use the limitations offered by apps like TikTok, although she admits that she often skips them. Mariana (medium risk) reports having used an app that turns reducing screen time into a game, as does Alejandro (low risk), who uses an application that blocks the phone according to time schedules, with a financial penalty for non-compliance. Then, Diana (medium risk) explains that due to her tendency to overuse social networks and the emotional impact this has on her, she has created a second Instagram account which she keeps secret and only uses to follow content that interests her.

The interviews also reveal several particular cases that are worth examining. Firstly, in the high-risk group, there is a minority group whose regulation strategies involve leaving the device with someone else. Ignacio tries to limit his access to his smartphone by leaving it in the locker at the gym or giving it to a friend while he is studying in the library. In the case of Carla and Daniela, the burden of regulation falls on their mothers by their own choice.

*(Interviewer): Were you worried about it in the end? Did you decide to do something?*

*(Daniela): Well, one time I noticed and I told my mum to take the phone away from me because I was under a lot of social pressure.*

*(Interviewer): And do you think it had an effect?*

*(Daniela): It helped a bit, but I fall back into it. (Daniela, high risk).*

Secondly, in the low-risk group, there are regulation strategies framed within deeper contexts of personal change. These cases shed light on the trajectories of participants between different levels of abuse, with personal contexts as the common thread. In the cases of Alejandro and María B. (both low-risk), the attempt to regulate smartphone use is framed in a context of personal change, preceded by what they describe as past periods of smartphone abuse. For Alejandro, personal change is related to a change in lifestyle habits linked to physical activity, rest or diet. For María B., however, personal change is linked to a break-up, returning to her parents’ house in her home town and starting to study to become a civil servant. In both cases, daily smartphone use is methodically scheduled, restricted to uses, such as listening to music or podcasts, which do not affect the development of activities such as studying or playing sports. María B. says that she always has her smartphone on work mode and only answers messages from her parents and brother. Alejandro, in turn, values his belonging to a Telegram community, in which digital regulation is linked to issues related to personal improvement.

The case of Iván (medium risk) can also be included in this category. Being immersed in a process of reconsidering his digital practices, he acquired an old model of mobile phone.

*(Iván): For example, I bought an old mobile phone, a BlackBerry, because in a way it’s good not to have the medium, not to have, for example, internet availability, or Instagram, or social networks, because I end up using it less, because... Well, in the end I use the internet—the new technologies—for the academic context, for example, but I don’t think that’s an addiction, I think it’s an adaptation. Then there is the addictive part that I am struggling to get rid of, the part that is not functional, so to speak. (Iván, medium risk).*

However, his case can also be linked to the emergence of two points to consider. In the first place, with the exception of a few cases such as Iván, the interviewees do not report it as part of their self-regulation strategies, yet it is striking that several interviewees from the medium-risk group (Mariana; Diana; Jorge; Jordi; and Javier) agree that they prefer using the computer to do work or school tasks. Secondly, although it was only mentioned in one case (Mariana), a practice that stands out is a sort of digital binge consumption, in which after a period of exams or intense activity, the person in question ‘rewards’ themselves with a whole day in bed consuming all kinds of digital content. This raises the interesting question as to what extent this type of binge consumption can be part of digital regulation practices.

### The language of addiction

The language of addiction completes the contrast between abuse practices and self-regulation strategies, connecting the narratives of the interviewees with their reflections on themselves within the framework of what we call the culture of addiction. This concept unites narratives linked to addiction in three complementary dimensions: addiction to digital technologies and its causalities; the language of addiction applied to others, and the language of addiction applied to oneself. The analysis reveals quite clear differences between the first two sub-codes and the third.

## Addiction to digital technologies and its causalities

More than half of the participants (15) expressed their views on this issue regardless of the level of risk. A small group, such as Julia, Basilio, and Javier, simply expressed a positive opinion on the existence of an addiction to digital technologies. Whereas a majority group specified the causality between technological and human factors. To the three participants (Daniela, Illen, and Alejandro) who believe that the problems of digital abuse are caused by individuals misusing the technology, we must add those cases (Antonio, Iván, and Estefanía) where, while recognizing the weight of the technological factor, they ultimately attribute responsibility to the uses made by each individual.

(*Estefanía): I think it’s more about the person, because the applications are already there and we already know what they were designed for and what they’re for, and I think it’s up to the person to decide how to use them. (Estefanía, low risk).*

Only five interviewees (Ignacio, Jordi, María S., María B., and Rafa), focus on the very nature of these technologies and their possible ‘addictiveness.’

*(María): Of course, it’s like a drug, you know, that you want to get that thing, or whatever it is that it’s giving you, because in fact at that moment that momentary happiness, that momentary pleasure—rather than happiness, momentary pleasure that you’re getting from watching what you like or this or that, you know? […] Of course, and that does influence focus, because what would you prefer, to be on your phone or studying something about history? (María S., medium risk).*

## The language of addiction applied to others

The second subcode was less present, with 9 of the interviewees (4 high risk, 3 medium risk, and 2 low risk) making comparisons between their smartphone use and what they perceived in their personal environments, pointing to the social prevalence of an addictive relationship. Three of them, Julia, Antonio, and Illen, stated that they saw ‘everyone equally hooked.’ Meanwhile, another four, Ignacio, Marcelo, Iván, and Mariana, went further, pointing out that the ‘normalization’ of this digital addiction prevents it from being perceived.

*(Iván): Well, I think it’s the typical paternalistic discourse of our parents, but on a friend’s level, as in general everyone follows the same dynamic, nobody is pestering you, because you have to be really hooked for-for someone to say anything at all with the way we are all using it. (Iván, medium risk).*

Only two, María B. and Estefanía (low risk), use this comparison to stress that they feel ‘less hooked’ than others.

## The language of addiction applied to oneself

The use of the language of addiction applied to themselves only emerges in the high- and medium-risk groups. Six of those belonging to the high-risk group (Antonio, Basilio, Carla, Daniela, Ignacio, and Julia) defined their relationship with the smartphone as an addiction. Both Antonio and Carla described this ‘addiction’ as ‘very strong.’ On the other hand, Basilio admitted to feeling ‘hooked on his phone,’ while Daniela pointed out that despite trying to control it, she ‘falls back into it.’ In the same vein, Joaquín compared his excessive use of the smartphone to smoking tobacco. The case of Marcelo is particularly interesting, as he defined himself as a potential addict and therefore claimed to be very cautious with his occasional consumption of cannabis, although his main concern lied in his inability to stop consuming pornography.

*(Marcelo): Well, seeing how often this happens to me, picking up my phone, using it so much every day and not knowing when to stop, the feeling I had when I didn’t have it... (Marcelo, high risk).*

The cases of Ignacio and Julia, meanwhile, are slightly different. Julia claimed to have been ‘hooked’ on some games that she accessed with her smartphone during lockdown, while Ignacio, a psychology graduate, defined himself as ‘hooked’ on what he described as a vicious circle of smartphone use.

*(Ignacio): Also, it’s a vicious circle, in that, maybe, you feel bad about your situation at the time and you start using your phone, and maybe you can’t stop and you start feeling worse too. (Ignacio, high risk).*

In the medium-risk group, 7 of the members (Illen, Iván, María S., and Sabrina) used the expression ‘being hooked’ to refer to their smartphone abuse. Sabrina in a more general sense (‘you get hooked and you can’t let go’), Illen, in relation to night-time use, and Iván metaphorically referring to withdrawal symptoms.

*(Iván): And so it’s like a self-imposed limitation that I’ve set for myself in order to... well, to control myself and be aware of the addiction it generates in you.*

*(Interviewer): Mmm.*

*(Iván): And so tomorrow I’ll switch to detox mode and feel what it’s like to be on withdrawal. (Iván, medium risk).*

As for Jordi and Javier, they emphasize the key role of scrolling in what they consider to be an addictive relationship with smartphones. Jordi refers to watching short videos on YouTube or TikTok, and how ‘staying put’ watching videos without being able to stop is a waste of time. Javier reported that at one point he had associated watching videos with smoking cannabis, and despite having quit smoking, he has ‘kept’ that habit.

## Discussion

The results of the study show common digital practices among the participants. Essentially the smartphone acts as an integrating device, incorporating communication and content consumption uses into everyday life through almost ritualized routines. It also integrates the sphere of personal intimacy with the digital environment. The interviewees live with the constant presence of the smartphone, which involves managing its influence of the device on their attention, the activities they engage in and their time organization. This management articulates what we have defined as abuse practices, self-regulation strategies, and the language of addiction, outlining a socio-cultural context in which individual experiences fit. This calls into question the arguments that highlight the heterogeneity of smartphone use (Griffioen et al., 2021), while calling for a complex understanding of social factors (Amiri & Dowran, 2020; Olivella-Cirici et al., 2023).

Faced with this digital tide present through the smartphone, those interviewed generate individual systems of regulation, the results of which range from a sense of control to real control. This corroborates the findings that point to the perception of a lack of control as the most recurrent result among different samples of young users (Biedermann et al., 2021; Yang et al., 2021; Ochs & Sauer, 2023). These systems constitute unstable balances (Schuster et al., 2023) between their digital practices and the perceived effects on their well-being, relationships, or obligations. Their effectiveness seems to be modulated by individual aspects such as maturity, personal situation, or the availability of free time. As well as by factors that are more social, such as family or institutional patterns of regulation, educational guidelines, or the significance of other activities. In relative terms, the result is visible in the level of risk, the opposite of which would be what some studies call critical awareness (Arrivillaga et al., 2022).

The analysis corroborates the widespread existence of smartphone abuse practices, crystallizing at certain times into problematic uses that are more or less discontinuous. The differences are subtle between the high- and medium-risk groups, and clearer with respect to the low-risk group. In the high-risk group, abuse practices are intertwined with constant questioning, which paradoxically seems oriented toward achieving a sense of control that justifies their continuation. This contrasts with the findings of the medium-risk group, which also has very present abuse practices, but these are limited to specific moments that include occasional digital binge consumption, and have a lesser effect on everyday activities. However, it is most evident in the low-risk group, in which there are hardly any abuse or self-control practices.

These observations are reflected in details such as the precise time estimates of use expressed by the members of the high-risk group, as well as in their perception of the smartphone as an almost inescapable distraction. Similarly, we see this in the way in which the members of the medium-risk group represent the daily influence of the smartphone as a temptation reserved for leisure time or as a distraction against which they must take precautions. This is also the case with self-regulation practices. Whereas among those in the high-risk group, the use of specific strategies is predominant, such as temporarily deleting some apps, and in the medium-risk group, we see more comprehensive strategies, which often involve relying on the digital technologies themselves. This is quite different for the low-risk group, in which, with some exceptions, there are no explicit attempts at self-regulation, as if they did not need it.

The link of these issues to self-perception emerges more clearly in the use of the language of addiction. Although only a minority of interviewees attributed the problems of digital abuse to the individuals themselves, the majority assumed that despite the influence of technological design, individuals themselves should be responsible for their own regulation. Similarly, a large proportion of those interviewed said they perceived others to be as or even more ‘hooked’ on their smartphones than themselves. The application of this ‘addictive perception’ of themselves under the term ‘being hooked’ mostly appeared among those interviewed who were at high and medium risk. On this point, the results concur with studies (Adorjan & Ricciardelli, 2021) that stress the coexistence of discourses on addiction among young people, with ironic positions regarding their agency with respect to technological designs. The use of these expressions is related to several global trends (Courtwright, 2019): first, the presence in conventional and digital media of confessional accounts by celebrities and influencers who narrate their supposed addiction to digital technologies and their rehabilitation; second, the emergence of a therapeutic industry offering digital detoxification treatments and programs, and the proliferation of apps focused on developing healthy habits, which link certain ideas of digital diet with elements of renewed interest in old ideas popularized as stoicism; and third, the perception that young people are a passively vulnerable group when they come into contact with the digital world (Vickery, 2017)

At a more specific level, the analysis reveals the theme of digital bingeing. Despite its sporadic presence, this has a structural resonance with our explanatory framework that would be interesting to explore in future. Digital bingeing exemplifies the practical complementarity between the intensive use and hyperpresence of the smartphone, with the management of this fact through self-questioning about the addictive or healthy nature of such practices (Hartogsohn & Vudka, 2023). In this framework, which turns the issue of abuse into a question of morality and willpower, bingeing takes an ambivalent meaning: as a form of gratification (Fullwood et al., 2017; Irimiás et al., 2021; Zarhin, 2024) resulting from having shown self-control, but also as an occasional weakness that show the need for such self-control strategies.

Comparisons with binge eating or alcohol drinking often explore the correlation between behaviours displaying addictive patterns (Grant et al., 2019; Wang et al., 2023; Bastos et al., 2024). These comparisons could also serve to show the extent of the problematization framework of addiction. These forms of bingeing have a regulatory function, an episodic and repetitive nature, and a ritualized organization. While the differences appear to be related to social regulation and the stigmatization or normalization of such consumption, the supposed greater involvement of design in digital technologies such as smartphones is nuanced if we consider advertising networks and other social aspects linked to public regulation as significative mediations.

The issue of digital bingeing enhances a key aspect of the culture of addiction, namely the imminent individualization of possible solutions. This is a key principle of neoliberal political models (Rose, 1998; Wacquant, 2012) and is also largely outlined in the self-regulation strategies described by the narratives. However, some narratives reveal signs of a less individualistic approach that would be worth exploring. Examples include the diversification of devices for specific uses or the imposition of restrictions on use in certain work and school environments. These regulatory principles link with the mapping of specific risk or protection factors in youth cultures of smartphone use (Cuesta Cambra & Herrero, 2014; Kiss et al., 2020) and highlight the need for techno-educational approaches and co-responsible regulation by the technology companies themselves (Gui et al., 2023). Unfortunately, in the local context of the study, this approach appears to stem more from the efforts of social groups associated with families than from political spheres.

Finally, in light of the public health implications, it is important to highlight the explanatory potential of personal trajectories and the need for longitudinal studies. Among the respondents, there were some interesting cases because their participation in this study matched periods of deep personal change, which explained their reconsideration of smartphone use management. In these cases, the self-regulation strategies can be understood as attempts to stabilize a change whose success is difficult to predict and may depend on external factors.

## Limitations

Although this study aims to show the cross-cutting presence of certain patterns among different age and risk groups, the data also point to nuances and differences whose significance could not be verified. In this regard, the qualitative analysis and some additional statistical tests (Chi-square tests) performed on the main variables and effect size metrics (contingency coefficient), suggest that the composition of the analyzed sample may be a possible limitation of this study. The analysis derived from the interviews might have been relatively different if there had been more respondents from the low-risk and lower age group (15–19 years). Further research on these groups could be a focus for future work.

## Competing interests

The authors declare no competing interests.

## Data Availability

Due to data ownership issues, the authors are not authorized to deposit the data in a public database. In case of interest, any researcher may contact the corresponding author of the article by e-mail.
